# Physicians as Fundraisers: Medical Philanthropy and the Doctor-Patient Relationship

**DOI:** 10.1371/journal.pmed.1001600

**Published:** 2014-02-11

**Authors:** Julian J. Z. Prokopetz, Lisa Soleymani Lehmann

**Affiliations:** 1 Yale Law School, New Haven, Connecticut, United States of America; 2 Department of Medicine, Brigham and Women's Hospital, Boston, Massachusetts, United States of America; 3 Division of Medical Ethics, Harvard Medical School, Boston, Massachusetts, United States of America

## Abstract

Lisa Lehmann and colleagues discuss how “grateful patient” programs that solicit donations from wealthy individuals who receive care may be problematic for physicians and propose policies that mitigate these risks.

*Please see later in the article for the Editors' Summary*

**Summary**

- American medical institutions commonly have “grateful patient” programs that solicit donations from wealthy individuals who receive care. Physicians are often encouraged to assist in these programs.
- Development efforts have intensified in recent years, and the increasing reliance on physician fundraisers risks blurring the lines between clinical care and fundraising. New changes to the US Health Insurance Portability and Accountability Act (HIPAA) Privacy Rule continue that trend by allowing development officials to access certain types of clinical information without patient consent.
- The practice of physicians fundraising from their own patients raises three main concerns: (1) undue pressure on patients to contribute, (2) possible expectations of preferential treatment from donors, and (3) concerns about patient confidentiality and trust.
- We propose that institutions voluntarily adopt development policies that mitigate these risks. Specifically, we recommend that patient consent be secured before development staff access patient information or physicians refer patients to the development office. We also recommend that physicians not directly solicit donations from their own patients.
- The concerns discussed here in the context of American grateful patient programs are relevant to similar patient fundraising efforts in other nations.

American medical institutions commonly solicit donations from wealthy individuals who receive care [1],[2]. Physicians are often encouraged to assist in these programs because their existing relationships afford them insights into each patient's clinical history, personality, and financial situation; experience has shown that physician involvement can increase the frequency and size of donations [1]–[3]. These types of development initiatives are often referred to as “grateful patient” programs, and there are indeed patients who find joy in giving back to the clinicians and institutions that have made a difference in their lives [3].

In the last several years, many institutions have responded to declining reimbursement rates and competitive research grant funding by intensifying their development programs [1],[4],[5]. These newer, more proactive approaches to identifying and soliciting potential donors risk blurring the lines between clinical care and fundraising, pushing the outer limits of gratitude and comfort for both patients and physicians. When the US Department of Health and Human Services (HHS) updated the Health Insurance Portability and Accountability Act (HIPAA) Privacy Rule in January 2013, they shifted the boundaries of permitted development activities by including both new protections for patients and new avenues for using clinical information for fundraising purposes. We propose that institutions voluntarily implement development policies that limit the use of patient information in order to safeguard the trust that forms the basis of the doctor-patient relationship.

While our discussion focuses on the specific example of the US HIPAA Privacy Rule, we note that the concerns raised in this article are relevant in other nations as well. Grateful patient programs also exist in countries where medical institutions are privately owned, such as Canada and Australia. Public health systems like the UK's obviate the need for hospital-based development efforts because all costs are borne by the government. However, the universities affiliated with academic health institutions have fundraising programs, as do professional organizations like the Royal Society of Medicine. In nations like Hungary, the public health system operates within a well-recognized economy of “informal payments” to doctors in order to secure faster or better care, raising many of the same concerns discussed here in the context of formal fundraising [6]. We note, however, that philanthropy from patients is somewhat less relevant to hospitals that primarily treat populations of low socioeconomic status.

## Background: Ethical Concerns with Physician Involvement in Fundraising

Some forms of physician participation in fundraising are fairly benign. Waiting rooms may feature brochures or posters highlighting fundraising initiatives, and patients who express interest in giving may be referred to development personnel.

Many development offices have begun taking a more proactive approach. Some have instituted streamlined protocols for regular wealth screenings of admitted patients, bedside visits to targeted potential donors, and VIP “concierge” programs with special amenities for patients who've given in the past [1],[7]. Institutions may also encourage physicians to notify the development office of potential donors among their patients. A 2010 randomized controlled trial revealed that training sessions can increase the number of physician-initiated referrals [2], and institutions can now hire private consulting firms that offer professional coaching services [8]. Some physicians go a step further and directly engage their patients in conversations about giving. They may begin by describing their clinical and research activities and then identify funding needs, or they may broach the subject of giving with wealthy patients who have recovered from a major procedure [3].

Physician participation in development activities can create tension between their roles as caregiver and fundraiser, potentially undermining the trust at the heart of the doctor-patient relationship. We identify three main concerns. First, patients may feel undue pressure to make a contribution if their caregiver is involved in making the appeal [4],[9]. They may worry that declining to give will damage their relationship with their physician or have an adverse effect on their treatment, particularly if the physician's care encompasses other family members or life-threatening conditions.

Second, there is the possibility that patients who decide to give may harbor expectations of preferential treatment. VIP concierge programs offer institutional comforts like private rooms [1],[4],[7], but large donors may hope for clinically oriented favors from their physician in the form of last-minute appointments, longer visits, or largesse with prescription medications that are not clinically indicated. Well-intentioned physicians may feel a desire to reciprocate patients' generosity, or feel pressure to accommodate donors' requests to protect the institution's financial interests [7].

Third, there are broader issues of privacy and trust [9]. Patients may be disquieted to learn that their physician assessed their financial situation and perhaps shared that information with non-medical staff in the hospital. They may worry that their doctor sees them as a purse rather than a person.

A recent qualitative survey found that many physicians with fundraising experience are sensitive to these concerns, though some physicians maintain that there are no ethical problems with fundraising from patients [7]. Physicians have also noted benefits to fundraising with their own patients, such as the increased motivation to provide excellent care and build strong personal relationships [3]. However, a physician's active role in the financial conversation means there is no longer a clear dividing line between the physician as health care provider and physician as fundraiser.

The American Medical Association (AMA) takes a hard line: in a 2004 opinion, the AMA Council on Ethical and Judicial Affairs recommended that “physicians should avoid directly soliciting their own patients, especially at the time of a clinical encounter” [9]. The AMA also noted that preserving patient confidentiality may require “permission from the patient [to] be obtained prior to divulging any information to third parties,” such as development officials [9]. Nonetheless, physicians continue to be actively involved in development work, often at the urging of development officials [1]–[3],[7].

## The New HIPAA Privacy Rule

In January 2013, the HHS promulgated significant changes to the HIPAA Privacy Rule. The new rule covers more entities, grants patients greater access to their own information, and expands the government's enforcement authority [10]. Less attention has been paid to the changed regulations on using patient information for fundraising purposes [11]. Some of the updates bolster patient protections; for example, we applaud the strengthened requirement that all development communications give patients a clear choice to opt out of future fundraising.

The new regulations also loosen the restrictions on access to clinical information. Formerly, development staff required a signed patient waiver to view anything other than basic demographic data, but they may now freely access three additional categories of information (45 C.F.R. § 164.514(f) (2013)) [11]. First, they may view basic patient outcome information. We support this change because it allows fundraising staff to avoid reaching out to patients who have died or experienced other severe health consequences.

What we find more troubling is that development staff may also view patients' department of service and treating physician, which further erodes the increasingly hazy barrier between clinical and fundraising activities. This may contribute to more frequent or more assertive requests from development to both patients and physicians, thereby heightening all three of the concerns outlined above.

Development staff may now proactively point physicians toward specific wealthy prospects among their patients, pressuring them to broach the subject of charitable giving or to personally reach out to patients with invitations to fundraising events. Additionally, physicians are now able to refer patients to development without their knowledge; under the prior rule, such a referral required signed patient authorization because the identity of the treating physician was protected information that would necessarily be disclosed.

These new possibilities raise special concerns for patient confidentiality and trust. Some departments and specialists reveal aspects of a patient's clinical history by simple association. Patients may be very sensitive to perceived invasions of privacy concerning care for conditions such as HIV, infertility, or mental health. Irrespective of the type of care, patients may be uncomfortable with the idea of physicians or development using information from clinical visits to support fundraising work without their knowledge or consent.

## Proposal: Voluntary Standards to Maintain Confidentiality and Trust

When HHS solicited public recommendations for the new Privacy Rule, it received many comments arguing that expanded access to patient information would “streamline…fundraising efforts and ensure that individuals were sent communications about campaigns that would be meaningful to their experiences” [11]. In the commentary accompanying the final rule, HHS noted that a “small minority of commenters” opposed the change because of “privacy concerns.” We agree with this latter group, and regret that their warning was not heeded.

As institutions encourage physicians to identify and engage potential donors among their patients, it is critical that we remain cognizant of the risks involved. In order to safeguard patient confidentiality and trust, we propose that institutions voluntarily adopt development policies that eschew the full depth of information available without consent under the new Privacy Rule.

Access to information on patient outcomes serves the important purpose of protecting patients and their families at times of vulnerability. Department and physician information, however, is primarily intended to aid targeted fundraising efforts. We recommend that, as under the prior Privacy Rule, development staff only access this information when authorized by the patient. Similarly, we recommend that physicians secure consent before referring one of their patients to development.

We note that the risks outlined above may be present when physicians engage in fundraising activities of any kind, including seeking permission to refer a patient to development. The only way to completely avoid these risks is to avoid fundraising at all. For physicians who choose to participate, we support the AMA's direction that physicians maintain a clear distinction between their development work and their clinical duties, and that they avoid direct solicitations of their own patients [9]. A strict policy of handling giving through formal development channels would attenuate all three kinds of risk by dissociating the physician from the actual financial request.

In countries like the United States, philanthropy is an important source of support for academic and clinical programs and a way for patients to show their gratitude. We offer these recommendations to support ethical fundraising efforts that gather financial support while protecting patients' wellbeing and preserving the integrity of the doctor-patient relationship.
